# On the founder effect in COVID-19 outbreaks – How many infected travelers may have started them all?

**DOI:** 10.1093/nsr/nwaa246

**Published:** 2020-09-24

**Authors:** Yongsen Ruan, Zhida Luo, Xiaolu Tang, Guanghao Li, Haijun Wen, Xionglei He, Xuemei Lu, Jian Lu, Chung-I Wu

**Affiliations:** State Key Laboratory of Biocontrol, School of Life Sciences, Sun Yat-sen University, Guangzhou 510275, China; State Key Laboratory of Biocontrol, School of Life Sciences, Sun Yat-sen University, Guangzhou 510275, China; State Key Laboratory of Protein and Plant Gene Research, Center for Bioinformatics, School of Life Sciences, Peking University, Beijing 100871, China; CAS Key Laboratory of Genomic and Precision Medicine, Beijing Institute of Genomics, Chinese Academy of Sciences, Beijing 100101, China; State Key Laboratory of Biocontrol, School of Life Sciences, Sun Yat-sen University, Guangzhou 510275, China; State Key Laboratory of Biocontrol, School of Life Sciences, Sun Yat-sen University, Guangzhou 510275, China; State Key Laboratory of Genetic Resources and Evolution, Kunming Institute of Zoology; Center for Excellence in Animal Evolution and Genetics, Chinese Academy of Sciences, Kunming 650223, China; State Key Laboratory of Protein and Plant Gene Research, Center for Bioinformatics, School of Life Sciences, Peking University, Beijing 100871, China

**Keywords:** SARS-CoV-2, COVID-19, population genetics, genetic drift, founder effect

## Abstract

How many incoming travelers [*I_0_* at time 0, equivalent to the “founders” in evolutionary genetics] infected with SARS-CoV-2 who visit or return to a region could have started the epidemic of that region? *I_0_* would be informative about the initiation and progression of the epidemics. To obtain *I_0_*, we analyze the genetic divergence among viral populations of different regions. By applying the “individual-output” model of genetic drift to the SARS-CoV-2 diversities, we obtain *I_0_* < 10, which could have been achieved by one infected traveler in a long-distance flight. The conclusion is robust regardless of the source population, the continuation of inputs (*I_t_* for t > 0) or the fitness of the variants. With such a tiny trickle of human movement igniting many outbreaks, the crucial stage of repressing an epidemic in any region should, therefore, be the very first sign of local contagion when positive cases first become identifiable. The implications of the highly “portable” epidemics, including their early evolution prior to any outbreak, are explored in the companion study (Ruan et al. 2020).

## Introduction

It is generally accepted that, in principle, a small number of infected people arriving in a new place could trigger an epidemic (if the basic reproduction number, $R_0$, is not too small [1-3]). The main issue is how many travelers *actually* started each epidemic. Here, "a new place" may mean a country, or a bordered

ORIGINAL UNEDITED MANUSCRIPT

region, within which the bulk of human interactions happen. Relative to the within-region movement, a bordered region is lightly connected to the rest of the world. Since the epidemic in any bordered region could have been started by one single infected traveler, or by 1,000 of them, we take the population genetic approach to analyzing the divergence among viral populations in relation to the "founder effect" [4].

We shall let $I_t$ be the amount of input at time $t$ (*i.e.*, the number of infected people coming in an uninfected region). The crucial number is $I_0$, *i.e.*, the first batch of input. The magnitude of $I_t$ is important in the public health practice. If $I_t$ has been large with continual input lasting for weeks, then a bordered region may be able to prevent the epidemic from being exported to (or being imported from) other regions, solely by restricting human movements out of (or into) its borders. On the other hand, if the epidemic in a region could be started with $I_0 < 10$ (and $I_{t>0} \sim 0$), then sealing off either emigration or immigration would not be effective in stopping a pandemic. Unless the bordered regions are maintaining zero infections, the danger would be coming mainly from within their borders. Here, we aim to infer $I_t$, in particular, in the early period of an epidemic.

In this study, we use a population genetic framework [5]. Because the focus is the stochastic differentiation among viral populations, epidemiological models generally do not cover such topics. The genetic drift formulation used here also permits the calculation of the extinction probability of the invasion of the virus ([5], Ruan *et al.* 2020). Epidemiological parameters, such as the number of uninfected individuals, the effects of quarantine and the development of immunity [6, 7], are not considered here as population differentiation takes place in the earliest stage of the invasion. During this stage, neither quarantine nor herd immunity has yet become a major factor in the outbreak.

**Theory –**

To estimate $I_t$, a conventional method is to inspect the changes in the population size of the viruses, $N_t$ [8, 9]. Viral population size corresponds to the number of infected individuals, assuming a viral clone in each person. Because $N_t$ is only weakly dependent on $I_t$, the conventional approach does not offer the resolution we seek for. It would be more informative to examine multiple populations for their differences in genetic polymorphism. The differences would depend strongly on $I_t$ at the very beginning of the epidemic (Fig. 1).

For studying population differentiation, the source population infecting the travelers needs to harbor genetic variants in non-trivial frequencies to yield informative data [10]. For example, Tang *et al.* [11] reported the existence of two lineages that are distinguishable by two SNPs, one being a Serine/Lysine (S/L) polymorphism. According to ref. 11, the S lineage accounts for ~30% and the L lineage accounts for ~70% among the 103 viral genomes they examined.

ORIGINAL UNEDITED MANUSCRIPT

For the ease of estimating $I_t$'s, the variants should ideally be neutral in fitness. Indeed, since variants under selection would have a short retention time in the population, SNPs are often neutral [12-14]. While the fitness differential between the L and S variants remains unclear, our simulations show that the estimation of $I_t$ is only weakly dependent on selection (see below).

The estimation of $I_0$ as well as $I_{t>0}$ are conducted for multiple viral populations, which should ideally originate from independent samples. Among these populations, we model their differentiation as a function of $I_t$. $I_t$ is not likely to be very large between regions (including the source) reachable only by air flight, each of which may carry at most a small number of infected passengers. As long as all extant populations are derived from the same source population, the estimates of $I_t$ are only weakly dependent on the actual genetic make-up of the source. For that reason, the source population needs not be known.

The hypothesis is that the viral populations seeded by the infected travelers have experienced strong fluctuation in gene frequency. This may happen at the beginning of the epidemic when $N_t$ is small. Soon afterwards, the fluctuation in gene frequencies would be quickly dampened as $N_t$ grows. The fluctuation in gene frequencies due to the random transmissions of genes is referred to as genetic drift [14, 15] or the founder effects [4]. The standard formulation of genetic drift by the Wright-Fisher model (or the alternative model of Moran [16]) is not applicable for tracking the viral population. Instead, we use the "individual output" model we previously proposed [5]. All models assume discrete generations. Based on the infection dynamics estimated in a recent study [17], we assume that each discrete generation is ~4 days. If we use a longer or shorter generation time, the outcome would be similar as long as the progeny production is calibrated with the generation time.

From one generation to the next, each individual produces $k$ "descendants" (or infects $k$ others) with the mean of $E(k)$ and the variance of $V(k)$. In the Wright-Fisher model, $k$ follows the Poisson distribution and $V(k)$ is tied to $E(k)$ [5]. In the "individual output" model, $k$ may follow any distribution, which is often measurable but not in any common form. $E(k)$ dictates the population growth, $N_t$, while $V(k)$ determines the fluctuation in $N_t$ and in gene frequency. We will attempt to obtain $E(k)$ and $V(k)$ from the empirical data and, for a comparison, will also allow $V(k) = E(k)$ to approximate the Wright-Fisher model. $N_t$ is a function of $E(k)$, $V(k)$ and $I_t$. Here, we assume $I_t$ to be a constant, hence, $I_t = I_0$ for all $t$'s. At time $T$,

$$E(N_T) = \sum_{t=0}^{T} I_t E(k)^{T-t} = I_0 \frac{E(k)^{T+1}-1}{E(k)-1} \quad \text{when } E(k) > 1 \qquad (1).$$

If $E(k)$ is not too small, $E(N_T)$ would depend mainly on $I_t$ of the first few generations. In fact, the results are often similar whether there is constant input or not (*i.e.*, $I_t = I_0$, or $I_t = 0$ when $t \geq 1$). In other words, Eq. (1') below would yield similar results as Eq. (1)

ORIGINAL UNEDITED MANUSCRIPT

$$E(N_T) = I_0 E(k)^T \quad (1').$$

With reasonable accuracy, $E(k)$ can be obtained from the growth trajectory of $N_t$ but $I_0$ has to be obtained by a different means. While many of the assumptions such as exponential growth and the constancy of $I_t$ may hold for only a few generations, most of our results depend primarily on the dynamics of the first few generations. The actual trajectory of each population would also depend on $V(k)$. Using the simpler Eq. (1'),

$$V(N_T) = I_0 V(k) E(k)^{T-1} \frac{E(k)^T - 1}{E(k) - 1} \text{ when } E(k)>1 \quad (2).$$

To obtain $I_0$ for Eqs. (1) or (1'), we have to model gene frequencies. Using the example of the S/L polymorphism, we let $X_T$ be the frequency of the L lineage at time $T$. If the fitness of the S and L type is the same, then

$$E(X_T) = E(X_{T-1}) = \ldots = E(X_0) \quad (3)$$

$$V(X_T) = \frac{V(k)}{E^2(k)} \frac{X_{T-1}(1 - X_{T-1})}{N_{T-1}} \quad (4)$$

where $X_0$ is the frequency in the source population. Eqs. (3) and (4) will need some modifications if we use Eq. (1), or if we consider the fitness difference between L and S. (see Supplement).

**Simulations** –

The actual realization of $X_T$ in each population is obtained by iteration described here. We assume two types of viruses (L type and S type; [11]). The relative fitness of L type to S type is 1+$s$ ($s$ = 0 represents no selection). In addition, there is a source population, in which the frequency of the L type is $X_0$. At generation $t$, there will be $I_t$ immigrants from the source population. $I_0$ is the founder population size. A parameter $T$ sets the time limit of immigration. Thus,

$$I_t = \begin{cases} 0, if\ t > T \\ I_0, if\ t \leq T \end{cases}$$

At generation $t$-1, the numbers of the L type and S type are $L_{t-1}$ and $S_{t-1}$ respectively. Also, $N_{t-1} = L_{t-1} + S_{t-1}$ and $X_{t-1} = L_{t-1}/N_{t-1}$. After one generation, there will be $I_t$ ($I_t = I_L + I_S$; $I_L$, $I_S$ are the numbers of L, S type, respectively) immigrants from the source population. In addition, $N_{t-1}$ will increase to $N_t$. Thus, at generation $t$, the numbers of the L and S type are

ORIGINAL UNEDITED MANUSCRIPT

$$L_t = I_L + \sum_{i=1}^{L_{t-1}} k_i$$

$$S_t = I_S + \sum_{j=1}^{S_{t-1}} k_j$$

where $k_i$ is the progeny number of the i-th individual of either type. The distribution of $k_i$ will be defined in the next section. If there is selection, the number of L type will change as follows:

$$L_t = (1+s)L_t$$

At generation $t$, the population size and the frequency of L type are

$$N_t = L_t + S_t$$
$$X_t = L_t / N_t$$

Based on the definition above, we simulate the stochastic changes of the viral population until it reaches the 20th generation (i.e. $t = 20$). Since genetic drift is negligible when $N_t$ is large (e.g. $>10^5$), we simulate the trajectory by a deterministic model when $N_t > 10^5$.

To quantify the population differentiation, we calculate the pairwise Fst values [14, 18], defined below. With $X_t$ and $Y_t$ for a pair of populations,

$$Fst = 1 - \frac{X_t(1-X_t) + Y_t(1-Y_t)}{2\bar{p}(1-\bar{p})} \quad (5)$$

where $\bar{p} = (X_t + Y_t)/2$. If Fst = 0, $X_t = Y_t$ and the two populations are identical in gene frequency. If Fst = 1, the two populations are maximally differentiated with $X_t = 0$ and $Y_t = 1$, or vice versa.

*Defining the parameter set ($I_0$, T, $X_0$, s) and the distribution of k*

We set $I_0$ = 1,5,10,20,50, 100 and $T$ = 0, 1, 2, 3, 20. Thus, ($I_0$, $T$) = (5, 3) means 5 travelers each generation for 4 generations, counting $T$ = 0 as the initial batch. We set $T$ in this range but will show that $T$ = 0, 1 or 20 hardly matters. The frequency of L lineage in source population is $X_0$ = 0.1, 0.3, 0.5, 0.7, 0.9. Again, the polymorphism frequency in the source population has turned out to have little effect on the differentiation among populations. In addition, we also set $s$ = 0, 0.1 to study the effect of selection. For each parameter set [$I_0$, $T$, $X_0$, $s$], we repeat the simulation 100 times.

ORIGINAL UNEDITED MANUSCRIPT

As stated, the conventional Wright-Fisher Model requires *k* (progeny number of an individual) to follow a Poisson distribution with *V(k) = E(k)*. Here, we assume the spread of virus to be associated with the social network, which usually follows the power law [19, 20]. Specifically, we let *k* follow the Zipf's law (a discrete power-law distribution; [21]):

$$P(k = i; c, M) = \frac{1/(i+1)^c}{H_{M,c}}, i = 0,1,\ldots,M-1$$

where $H_{M,c} = \sum_{m=1}^{M} 1/m^c$. The mean and variance of *k* are

$$E(k) = \frac{H_{M,c-1}}{H_{M,c}} - 1 \qquad (6)$$

$$V(k) = \frac{H_{M,c-2}}{H_{M,c}} - \frac{H_{M,c-1}^2}{H_{M,c}^2} \quad (7)$$

The estimate of the basic reproduction number ($R_0$) of SARS-CoV-2 ranges from 1.4 to 6.5 [22-24]. Here, we focus on the early phase of the viral population growth by using $R_0 = 6.5$. The relationship between *E(k)* and $R_0$ is as follows:

$$N_t = N_0 R_0^{t/\tau} = N_0 E(k)^{t/G} \qquad (8)$$

where $\tau$ being the serial interval, *G* being the generation time. And $\tau$ is estimated to be ~5 days [23, 25] and *G* being 4 days [17]. Then $E(k) = R_0^{G/\tau} = 4.5$. (Note that *E(k)* would become smaller with smaller *G*, as stated above.) To have *k* follow the power law with *E(k)* = 4.5, we assume *M* = 30 and *c* = 1.3 in Eq. (6), which yields *V(k)* = 45. As noted in Chen YX *et al.* [5], the strength of genetic drift depends mainly on *E(k)* and *V(k)* rather than the actual distribution. By setting *V(k)* so large, we ensure that the $I_0$ estimate would be on the high side (see Discussion).

**Data** –

The estimation of $I_0$ as well as $I_{t>0}$ should be done on multiple viral populations that are independent samples from the same source. If they are not fully independent, then our estimates of $I_t$ would be conservative (i.e., over-estimation) since any exchange between populations should reduce the divergence.

Here, we generate two sets of data as shown in Table 1. In the hypothetical Set I, the gene frequency is taken from a normal distribution with the mean of 0.7 and standard deviation of 0.06. The mean is close to the average frequency of the L type in this pandemic. Set I is generated to show the possible pattern of population divergence if $I_0$ is in the hundreds.

ORIGINAL UNEDITED MANUSCRIPT

In Set II, we assign gene frequencies to 10 populations in Table 1 using the reported frequencies as a guide (GISAID (https://www.gisaid.org/); see Supplement). These 10 populations, distributed among 4 continents, are as likely to be independently derived as we could ascertain. The choice is based on three criteria: 1) the geography of the countries/regions and the distance between them; 2) the timing of the documented onset of the epidemic; 3) the abundance of DNA sequences. We consult the frequencies in samples collected before late March 2020, corresponding roughly to $G_8$ in Fig. 1. Due to the rapidly changing data reporting (GISAID [26]), the frequency profile of Set II is plausibly realistic as reported in mid-April (see Supplement). Readers with access to the more up-to-date data can compare the new observations with the theoretical results to improve the estimation of $I_t$.

**Comparisons between simulations and data –**

In Fig. 2, the Fst distributions based on the datasets of Table 1 are presented. The two very different distributions would be informative when compared with the simulated distributions.

Given the large number of possible combinations of parameters, [$I_0$, $T$, $X_0$, $s$], the task could have been daunting. Fortunately, all but one parameter would have little impact on the results. In Fig. 3A – 3D, only $X_0$ varies and the Fst distributions are very similar. The simplest explanation is that small populations would all deviate from the initial frequency, $X_0$, regardless of where it is. Fig. 3E-3H show that the results do not depend strongly on $T$ either, $T$ being the time duration of traveler input. Since, at $T = 2$, the number of infected individuals would have swelled by 20-fold without any new input, the results are not little affected by later inputs.

In Fig. 4, the value of $I_0$ is varied from 10, 50 to 100. Here, we assume no travelers arriving after the first generation because their contributions, as shown in Fig. 3E-3H, are insubstantial. The left panels (Fig. 4A, 4C, 4E) show the trajectories of 100 populations, which diverge in the first generation or two and then evolve steadily as the population size increases. On the right panels are the comparisons between the simulated and "observed" distributions; the latter being those based on Dataset II (Fig. 4B and 4D) or Dataset I (Fig. 4F). It's clear that the expected distribution of Fst is very sensitive to $I_t$ ($I_0$, in particular). The more realistic Dataset II can only be explained by $I_0 \leq 10$ whereas the artificial Dataset I would agree with $I_0 \geq 100$. We should note that the extensive survey of parameter space (see Supplement) shows the conclusion of $I_0 \leq 10$ to be robust.

In the results presented above, L and S lineages are assumed neutral. Intuitively, selection should drive the trajectories to converge and the left panels (Fig. 5A, 5C and 5E) do show that trend. If we let the L type enjoy a 10% selective advantage, the results of Fig. 5 still indicate $I_0 < 10$. Note that the range of $I_0$ spans a

ORIGINAL UNEDITED MANUSCRIPT

smaller range in Fig. 5 than that in Fig. 4. In other words, with selection, the estimated $I_0$ should be even smaller than indicated above.

The simulation results are based on the distribution of $k$ that follows the power law (see Eqs. 6 and 7) with $V(k) \sim 10E(k)$. Such a large $V(k)$ means that the genetic drift would be very large, requiring large $I_t$'s to reduce the drift. It is hence interesting that, even under such stringent conditions, $I_0$ is still < 10. In the Supplement, we show that the estimated value of $I_0$ would be substantially lower if we use the Poisson distribution of k, associated with the conventional Wright-Fisher model. With $V(k) = E(K)$, $I_0$ would be 2-4. Hence, the conclusion presented in this section is robust.

**Inference of parameters [$I_0$, $T$, $X_0$, $s$]**

In the last section, we present a range of parameter values that yield the expected population divergence for a comparison with the data. To corroborate the visual comparisons, we also carry out formal inferences using the ABC (Approximate Bayesian Computation) procedure on Dataset II (see Supplement). The results of Fig. S3 and S4 indeed confirm the visual impression as the observed divergence is sensitive only to $I_0$, but not to the variation in $T$, $X_0$ and $s$. The formal inference of $I_0$ at 2-5 is even smaller than the visual impression would suggest. Finally, this analysis considers only the number of infections that can spread the virus further. A more extensive model that incorporates the development of symptoms, the border control and the local quarantine, all of which may contribute to the suppression of the epidemics, will be presented later (Ruan *et al.* 2020b).

**Discussion**

In the theory of genetic drift [5], even a hundred infected travelers from a source viral population would give rise to a fairly uniform level of genetic polymorphism among bordered regions. In contrast, the reported data indicate substantial divergence among countries (GISAID (https://www.gisaid.org/); see Supplement). Dataset II of Table 1 is realistic in this respect. The divergent polymorphisms across countries depend mainly on a critical parameter – the size of the first cohort arriving in a country, $I_0$, which is estimated to be < 10. The number may in fact be smaller than it seems since a long distance flight carrying one single infected but symptomless patient could infect this many people, all of whom being without symptom upon arrival [27].

*On the robustness of the estimation*

In Figs. 3 and 5, we show that, despite the complexity of the model with many parameters, none of them [$T$, $X_0$, $s$], except $I_0$, plays a significant role in the divergence among viral populations. As discussed in conjunction with Fig. 3, the distribution of $k$ does not matter either. In fact, in the standard Wright-Fisher model, the estimate of $I_0$ would be < 5. It is also noted that the $E(k)$ and $V(k)$ values used is for a generation

ORIGINAL UNEDITED MANUSCRIPT

time of 4 days. For a shorter generation time, the values would be correspondingly smaller and the results should be similar.

The model also assumes that each population is an independent sample of the source population. Since all populations are likely to exchange some individuals due to travelling, the actual divergence among populations would be even smaller than simulated. In other words, to attain the observed level of divergence, $I_0$ would have to be even smaller than estimated. Considering all these variables, we believe the conclusion of $I_0 < 10$ to be robust. In the analysis of regional divergence, the results would depend strongly on the smaller $I_0$ of the two regions being compared. Hence, the assumption of the same $I_0$ among all regions would be a reasonable one.

*Subsequent viral evolution after arriving in a new continent*

While our focus is on the divergence in the first few generations, we now briefly discuss the subsequent evolution after this initial critical period. The primary lineage delineation, the S/L polymorphism defined by two SNPs [11], have many subtypes (see Supplement and Table S1 for details). For example, the western European countries including Italy, Switzerland, Germany and Belgium are predominantly of the L type with a similar abundance in the L2 subtype. In contrast, while Japan is also predominantly of the L type, it has mainly the L1 subtype. This contrast suggests that Japan may represent an independent sample from the western European samples, which have likely been spreading regionally after the initial seeding. Another example is the S1 and S2 subtypes, which differentiate between the samples from China and the west coasts of the US.

These patterns suggest that, after the initial seeding, each major region or continent has been evolving along an independent path. Since the initial seeding may be extremely difficult to prevent, the onus is to suppress the regional spread. The analyses of the subtypes in Asia, Australia, and various parts of North America would offer additional details of the spread of the virus, as has been done recently [28-31]. These details are beyond the scope of this study that focuses on the early stages of the viral spread.

*Implications*

The analysis suggests that the COVID-19 epidemic in each region surveyed was likely started by a very small number of travelers [$I_0 < 10$]. With such a tiny trickle of human movement, it would have been very inefficient for any region to prevent infected individuals from exporting an epidemic to (or importing it from) other places. For that reason, the crucial stage of repressing an epidemic in any region should be the very first sign of local contagion.

Finally, due to the "portability" of COVID-19, each epidemic, including the first one on record, could have easily been imported. Where then did all these epidemics begin? While the interest in the "origin" is

ORIGINAL UNEDITED MANUSCRIPT

intense, we suggest the question be broadened as "the origin and early evolution" of SARS-CoV-2. The latter implies a process whereas the former seems to mean a single time point. The process of early evolution may have stretched over different regions in a long time-span and involved multiple host species. Like many other evolutionary questions on origin, we suggest the question be phrased as the early evolution of SARS-CoV-2, rather than be about the "origin". The former implies a process whereas the latter seems to mean a single time point. This distinction is important as seen in the debates on the "origin" of dogs [32, 33] and new species in novel environments [34]. By compressing a process into a simple "origin", we may be asking a false question about, say, "the first dog" or "the first patient". The possible early evolution of SARS-CoV-2 is addressed in the companion study (Ruan *et al.* 2020).

**Acknowledgements**

We thank all those who have contributed sequences to the GISAID (Global Initiative on Sharing All Influenza Data) database (https://www.gisaid.org/). Acknowledgements and detailed information of the genome sequences we consulted for this study are given in Table S2. The actual data fitted to our model were computationally generated (see the main text). We thank Drs. Yaping Zhang, Suhua Shi, Weiwei Zhai, Jianzhi George Zhang, Bingjie Chen, Zheng Hu and Jindong Zhao for comments on and suggestions for this manuscript.

**Funding**

This work was supported by grants from the Ministry of Science and Technology of the People's Republic of China (No. 2020YFC0847000) to H.W., X.L. and J.L. This work was funded by Guangdong Basic and Applied Basic Research Foundation (2020B1515020030, 2019A1515010708), National Key Research and Development Project (2020YFC0847000), and CAS "Light of West China" Program.

**Competing interests**

The authors declare no competing interest.

**Table 1 Two computationally-generated datasets for the frequency of the L lineage**

| Region | A1 | A2 | A3 | B1 | C1 | C2 | C3 | D1 | D2* | D3 |
|---|---|---|---|---|---|---|---|---|---|---|
| **I (hypo-thetical)** | 0.73 | 0.70 | 0.71 | 0.64 | 0.75 | 0.61 | 0.70 | 0.74 | 0.82 | 0.69 |
| **II (realistic)** | 0.70 | 0.95 | 0.40 | 0.80 | 1.00 | 0.99 | 0.50 | 0.33 | 0.95 | 0.67 |

Notes –

1) Each letter in the region code indicates a separate continent and the number indicates a country or region.

2) D2* could have been derived from C1 or C2 due to the inter-continental travel pattern. Its exclusion from the analysis would increase the spread of Fst in Figs. 2 -4 and would thus lead to a smaller $I_0$ estimate.

ORIGINAL UNEDITED MANUSCRIPT

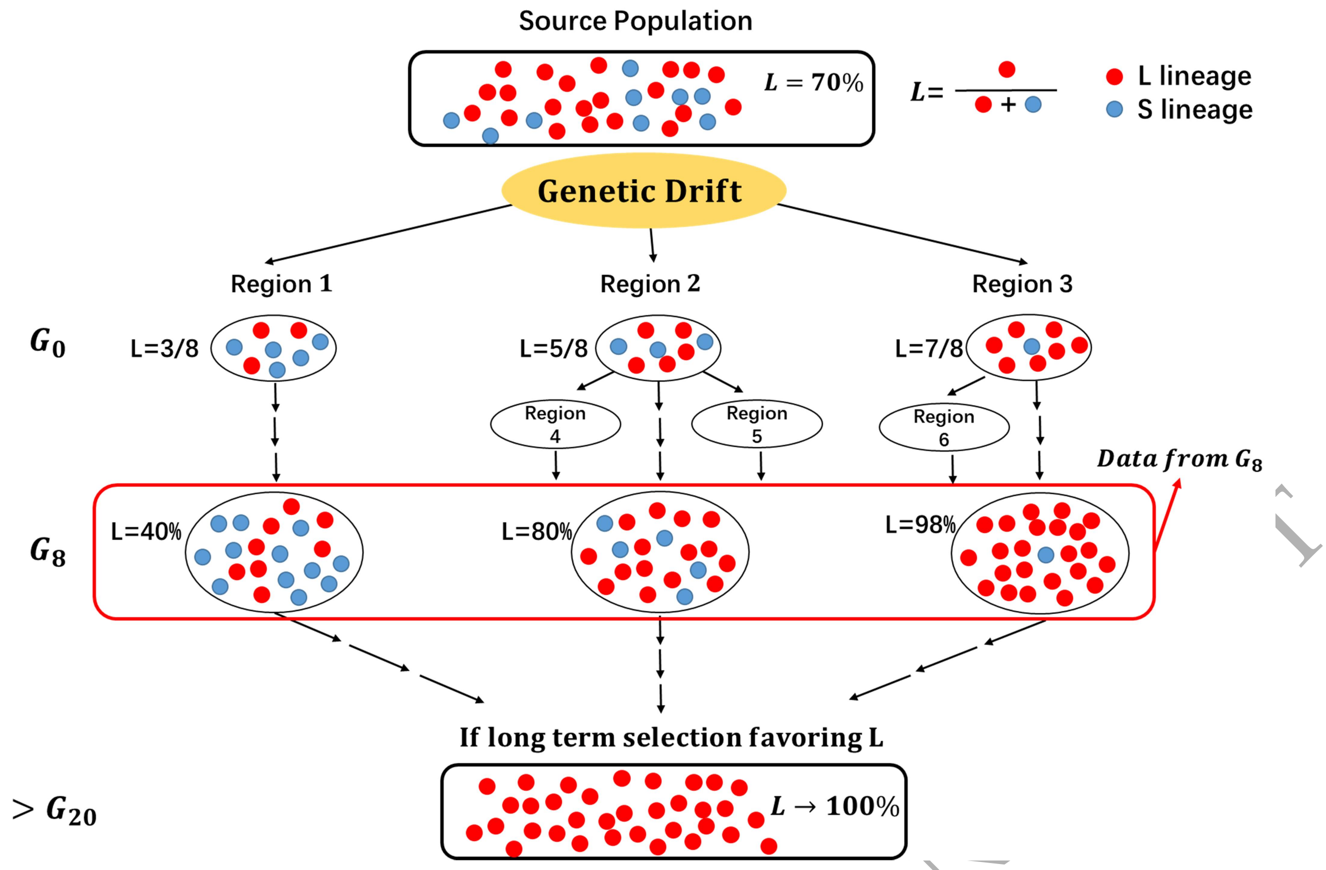

**Fig. 1 Schematic diagram of viral population divergence among regions.** In G0 (generation 0), $I_0 = 8$ and infected individuals arrive in regions 1 – 3 with 3, 5 and 7 of them, respectively, carrying the L-type virus. In the beginning, genetic drift is particularly strong and the frequency of L fluctuates as modeled by the Branching Process. G8 is about 5 weeks after the first arrival when the data are collected. After G8, the fluctuation is greatly dampened due to the large population size. In the later stage (after G20), even weak selection could drive gene frequency toward the fixation of the more contagious genotype. Regions 4 – 6 are not independent samples and are not included in the analysis (see Data of the main text).

ORIGINAL UNEDITED MANUSCRIPT

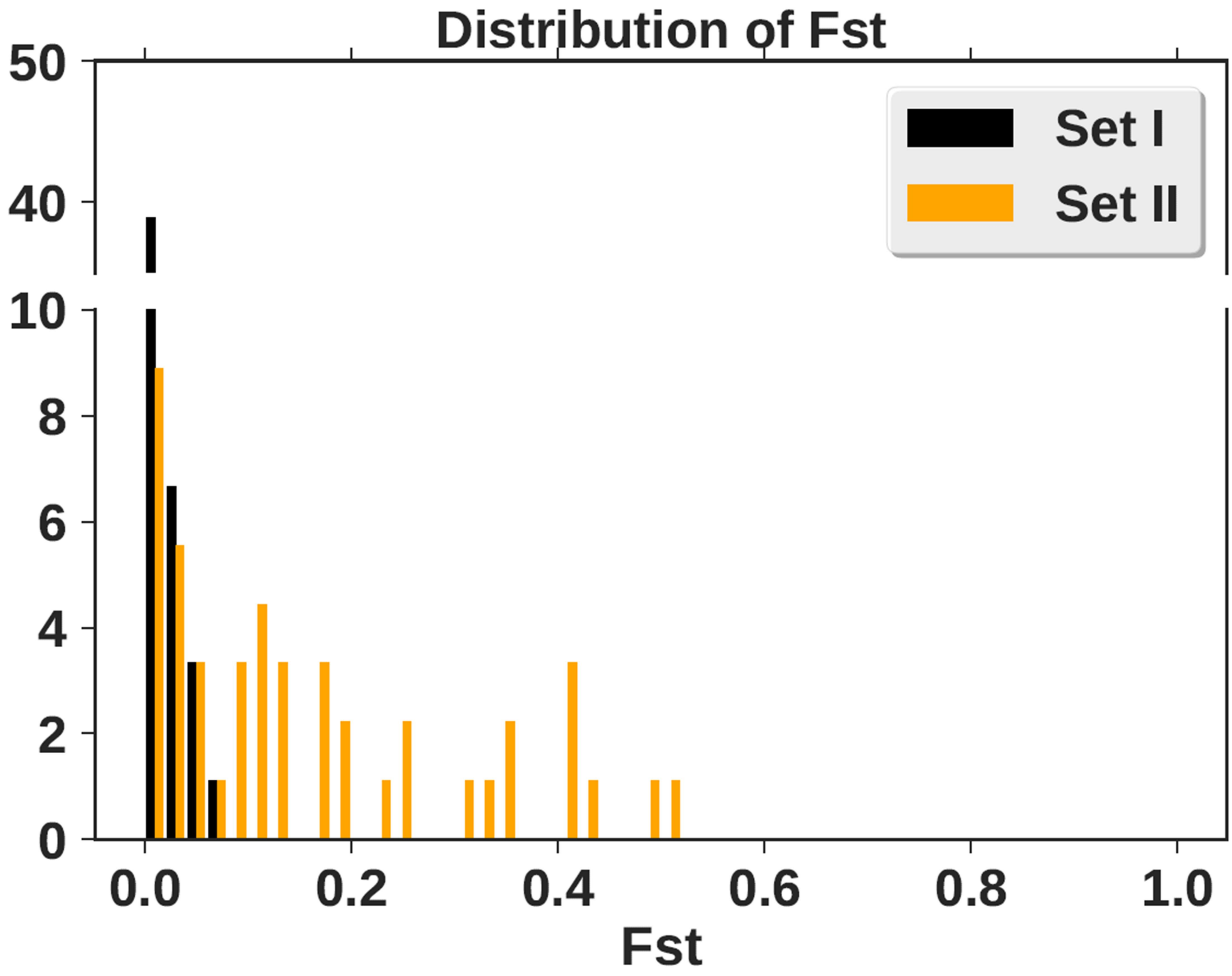

**Fig. 2 The pairwise Fst distribution from the two datasets of Table 1.** Fst is calculated by Eq. (5). Given 10 regions, there are 45 (=10x9/2) pairwise comparisons.

ORIGINAL UNEDITED

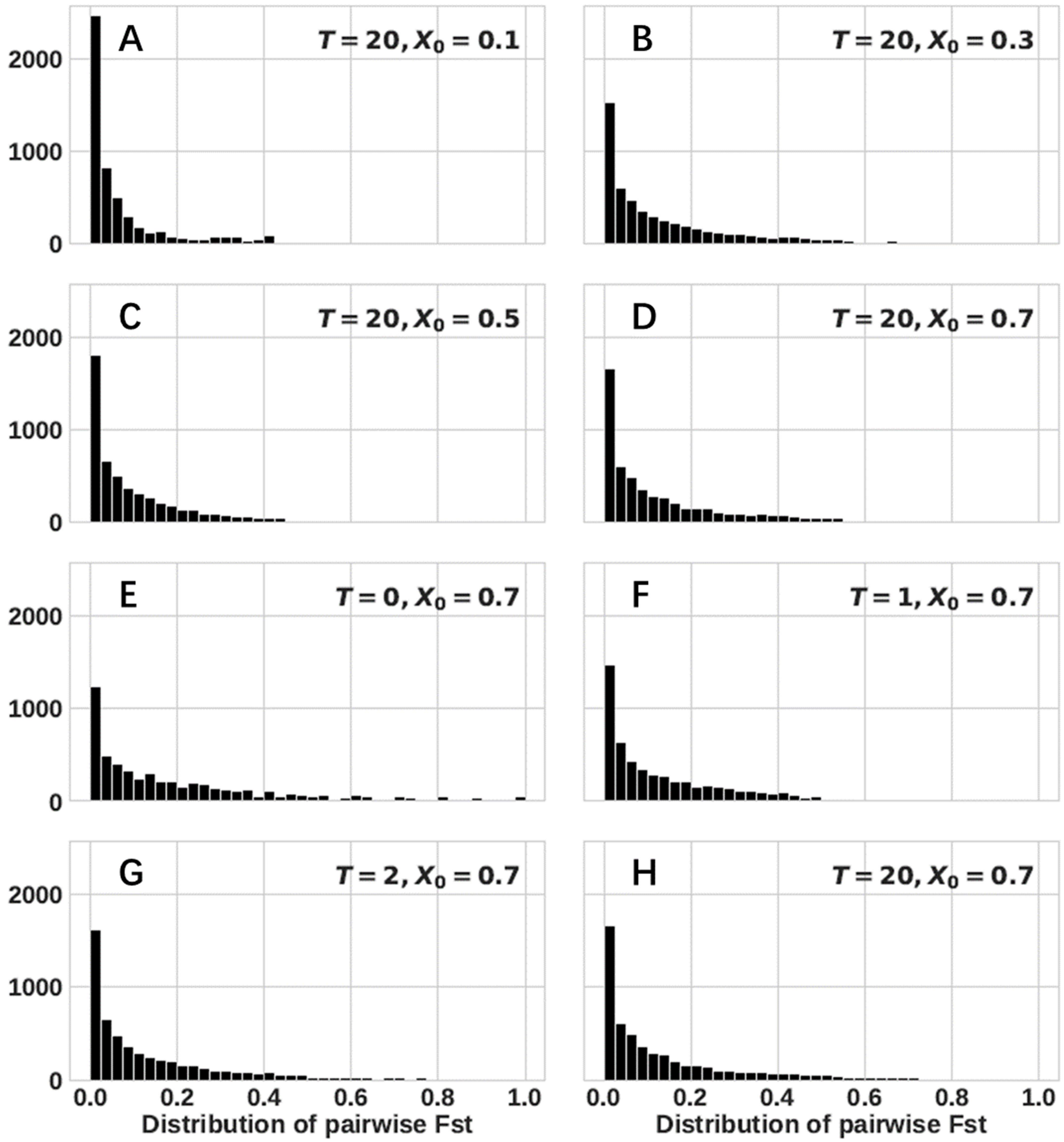

**Fig. 3 The Fst distribution at G20.** For each parameter set of [$I_0$, $T$, $X_0$, $s$], we repeat the simulations 100 times. For all panels, $I_0 = 10$, $s = 0$. Panel A-D, $T = 20$ and $X_0$ ranges from 0.1 to 0.7. Panel E-H, $X_0 = 0.7$, $T$ ranges from 0 to 20. These 8 panels show that neither $X_0$ nor $T$ would impact the Fst distribution much.

ORIGINAL

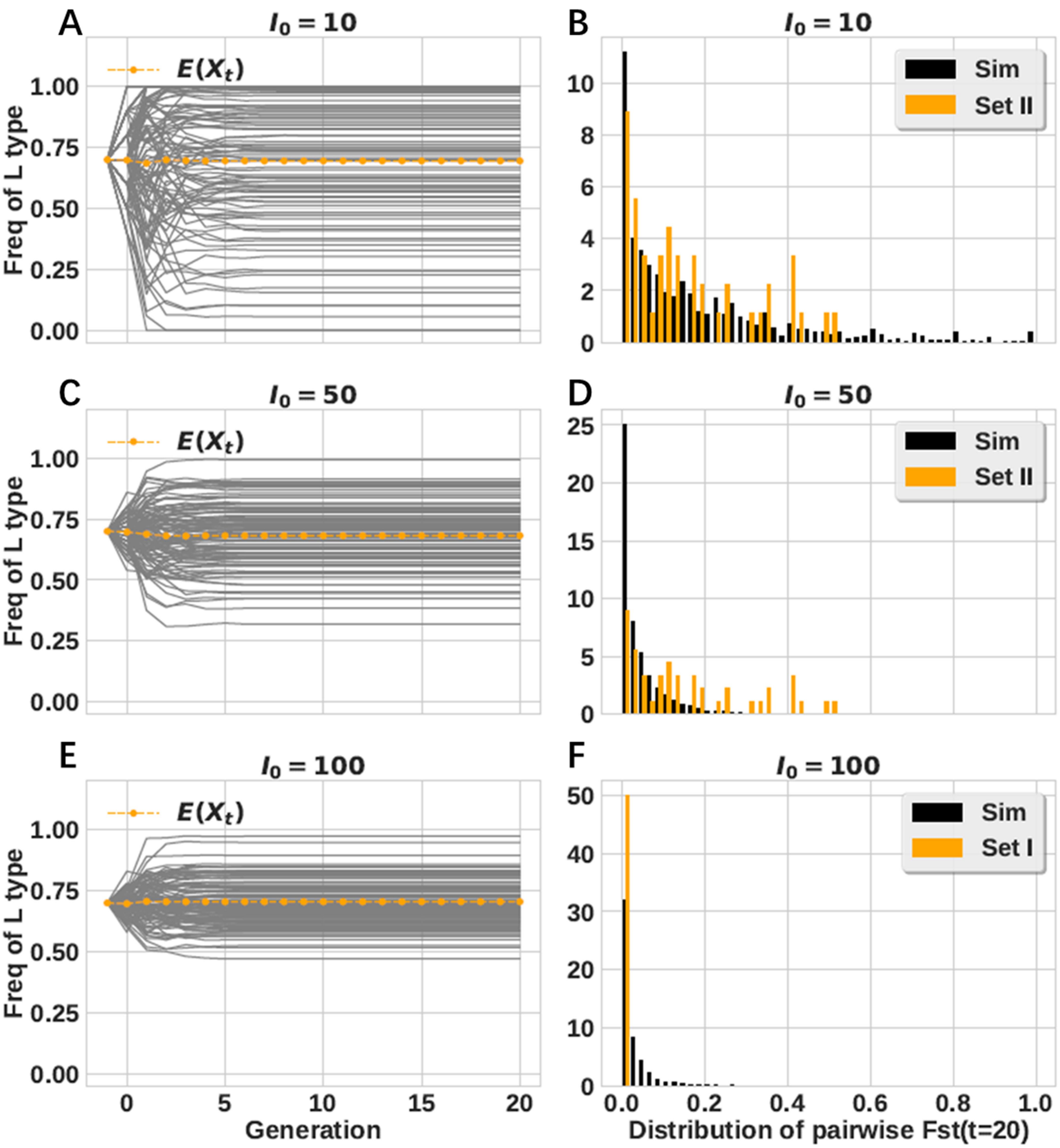

**Fig. 4 Comparisons between data and simulations with various $I_0$ values.** For all panels, $s = 0$ (no selection), $X_0 = 0.7$, and $T = 0$. The value of $I_0$ is shown next to each panel. Panel (A, C, E): the frequency of L type over time (100 repeats), the average is showed by the orange dotted line. Panel (B, D): Fst distribution; the simulation results are in black and the distributions from Dataset II (realistic data) are in orange. Panel (F): Like panels B and D but the Dataset I is used.

ORIGINAL

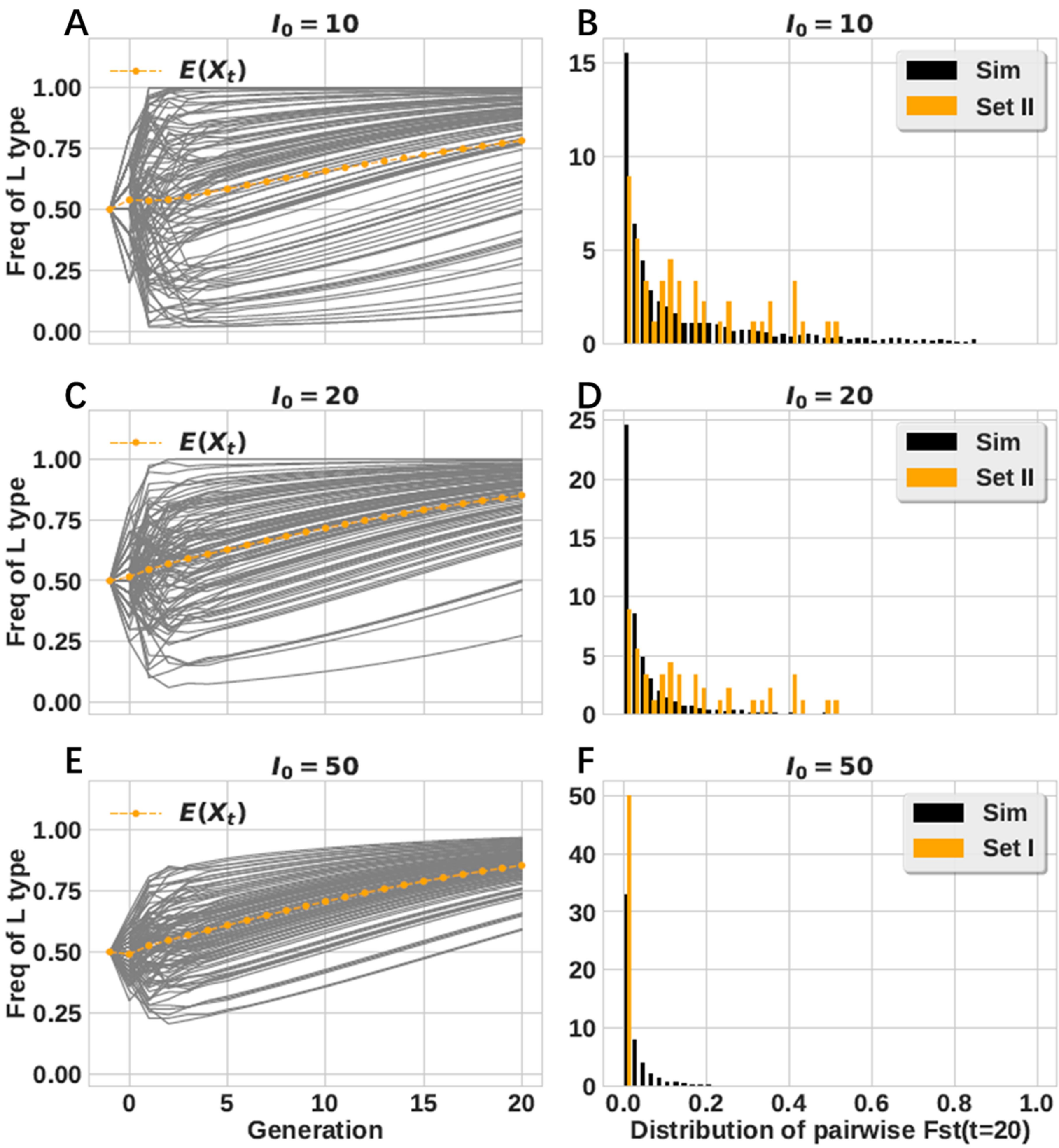

**Fig. 5** Same as Fig. 4 except that i) $s$ = 0.1 with selection favoring the L type; ii) $X_0$ = 0.5 so the L type would not reach fixation so quickly.

ORIGINAL
